# Microencapsulation and *in situ* incubation methodology for the cultivation of marine bacteria

**DOI:** 10.3389/fmicb.2022.958660

**Published:** 2022-08-22

**Authors:** Emily Pope, Christopher Cartmell, Bradley Haltli, Ali Ahmadi, Russell G. Kerr

**Affiliations:** ^1^Department of Biomedical Science, University of Prince Edward Island, Charlottetown, PE, Canada; ^2^Department of Chemistry, University of Prince Edward Island, Charlottetown, PE, Canada; ^3^Nautilus Biosciences Croda, Charlottetown, PE, Canada; ^4^Faculty of Sustainable Design Engineering, University of Prince Edward Island, Charlottetown, PE, Canada; ^5^Department of Mechanical Engineering, École de technologie supérieure (ÉTS), Montreal, QC, Canada

**Keywords:** microencapsulation, marine bacteria, microfluidics, biodiversity, microbiome, natural products

## Abstract

Environmental microorganisms are important sources of biotechnology innovations; however, the discovery process is hampered by the inability to culture the overwhelming majority of microbes. To drive the discovery of new biotechnology products from previously unculturable microbes, several methods such as modification of media composition, incubation conditions, single-cell isolation, and *in situ* incubation, have been employed to improve microbial recovery from environmental samples. To improve microbial recovery, we examined the effect of microencapsulation followed by *in situ* incubation on the abundance, viability, and diversity of bacteria recovered from marine sediment. Bacteria from marine sediment samples were resuspended or encapsulated in agarose and half of each sample was directly plated on agar and the other half inserted into modified Slyde-A-Lyzer™ dialysis cassettes. The cassettes were incubated in their natural environment (*in situ*) for a week, after which they were retrieved, and the contents plated. Colony counts indicated that bacterial abundance increased during *in situ* incubation and that cell density was significantly higher in cassettes containing non-encapsulated sediment bacteria. Assessment of viability indicated that a higher proportion of cells in encapsulated samples were viable at the end of the incubation period, suggesting that agarose encapsulation promoted higher cell viability during *in situ* incubation. One hundred and 46 isolates were purified from the study (32–38 from each treatment) to assess the effect of the four treatments on cultivable bacterial diversity. In total, 58 operational taxonomic units (OTUs) were identified using a 99% 16S rRNA gene sequence identity threshold. The results indicated that encapsulation recovered greater bacterial diversity from the sediment than simple resuspension (41 vs. 31 OTUs, respectively). While the cultivable bacterial diversity decreased by 43%–48% after *in situ* incubation, difficult-to-culture (*Verrucomicrobia*) and obligate marine (*Pseudoalteromonas*) taxa were only recovered after *in situ* incubation. These results suggest that agarose encapsulation coupled with *in situ* incubation in commercially available, low-cost, diffusion chambers facilitates the cultivation and improved recovery of bacteria from marine sediments. This study provides another tool that microbiologists can use to access microbial dark matter for environmental, biotechnology bioprospecting.

## Introduction

Environmental microorganisms are the source of a myriad of biotechnological products that have a substantial impact on society. These products range from enzymes that improve the effectiveness of laundry detergents to life-saving antibiotics (Kumar et al., 2008; Bérdy, 2012). The ability to culture a truly representative selection of microbes from environmental samples represents a seminal challenge within the fields of environmental microbiology and natural product discovery. As a result of this elusive microbial dark matter, the true biodiversity of the microbial world remains inaccurately represented by standard bacterial culture to date (Dias et al., 2012). This phenomenon has been named the “Great Plate Count Anomaly” and describes the discrepancy between the diversity of microbes observed in an environmental sample microscopically or detected *via* culture-independent methods, and those that can be cultured in the laboratory. Depending on the habitat, the proportion of microbes that are cultivable using standard culture techniques is estimated to be ~1% (Roberts and Caserio, 1977; Butler, 2004; Kinghorn et al., 2009; Krause and Tobin, 2013; Glatstein et al., 2014; Montinari et al., 2019). Over 100,000 bioactive natural products have been isolated from cultured microorganisms (Bérdy, 2012); thus, unculturable bacteria represent a significant untapped resource for biotechnology bioprospecting (Lewis and Elvin-Lewis, 2003; Cragg and Newman, 2005; Zhang and Demain, 2005). Natural product discovery reached its peak during the twentieth century with the discovery of numerous antibacterial, antifungal, antiviral, immunosuppressant, antiparasitic, anticholesterolemic, and anticancer drugs, however, the discovery rate of new natural product-derived drugs has significantly decreased over the past three decades. Development of new strategies to access the natural product repertoire of uncultured bacteria represents an attractive strategy to reinvigorate natural product discovery (Yazdankhah et al., 2013; Bbosa et al., 2014; Katz and Baltz, 2016; Newman and Cragg, 2016).

To counteract the “Great Plate Count Anomaly” and ultimately assist in the discovery of novel natural products, several strategies have been devised. Careful modifications of cultivation conditions by altering the media composition (e.g., tailoring salinity and pH and addition of habitat-derived extracts) or changing growth conditions (e.g., incubation time, temperature, pressure, salinity, or oxygen levels) have led to the successful cultivation of several new taxa of bacteria (Schut et al., 1993; Vishnivetskaya et al., 2000; Bruns et al., 2002; Sait et al., 2006; Omsland et al., 2009; Pham and Kim, 2012; Lok, 2015; Kawasaki and Kamagata, 2017; Kato et al., 2018); however, limited *a priori* knowledge of the metabolic requirements of diverse taxa limits the use of these strategies. Single-cell isolation strategies have also resulted in the cultivation of unique taxa, however, this methodology does not allow for cell-to-cell communication, which is required for the growth of some bacteria (Perbal, 2003). Cellular communication often involves the transmission of signal molecules, such as auto-inducers or peptides, which may play a role in cell growth (Perbal, 2003). To account for the dependence of some taxa on interactions or signaling from other organisms, co-culturing or community culturing techniques have been developed. These techniques use one or more helper bacteria to promote the growth of dependent bacteria (Pham and Kim, 2012). This approach is complicated by the large number of combinations that may be used, as well as the differing growth requirements and uneven growth rates of bacterial combinations (Nai and Meyer, 2018).

To further improve the recovery of “difficult-to-culture” microorganisms, *in situ* incubation has emerged as an effective technique. This approach is typically combined with single-cell isolation, and then the isolated cells are exposed to the natural chemical and nutritional conditions of their original habitat to promote replication, after which the cells are more amenable to cultivation in the laboratory (Gerwick and Moore, 2012; Jacoby, 2017). This technique has evolved from low throughput, large hollow diffusion chambers constructed of washers with permeable membranes forming the boundary between the chamber and the environments to the Ichip, which miniaturized this concept and encapsulated bacteria in 384 small agar plugs sandwiched between permeable membranes (Aoi et al., 2009; Nichols et al., 2010; Berdy et al., 2017). These devices allow diffusion of nutrients and other signals into the chamber while preventing migration of bacterial cells into, or out of the chamber. The primary advantage of these systems is that they provide the numerous environmental conditions necessary for bacterial growth that are difficult to identify and provide under laboratory settings (Kong et al., 2010; Gerwick and Moore, 2012); however, as the cells are isolated they do not allow for nutrient and/or signal exchange between members of a microbial community, which may limit the ability to culture taxa that require these interactions (Blunt et al., 2018). We recently reported the use of a modified Ichip for use in sponges which led to the isolation of previously uncultured bacteria and the subsequent identification of a new tyrosine derivative (MacIntyre et al., 2019).

In this study, we set out to build upon the diffusion chamber concept. We encapsulated bacteria extracted from marine sediment in agarose-based micro beads and then inserted the beads into a modified dialysis cassette, which was then incubated *in situ* for 1 week. To evaluate the effect of this approach on the recoverable bacterial diversity we compared the bacterial diversity obtained from this method to: (1) bacteria dislodged from the sediment, inserted in the dialysis cassette, and incubated *in situ*, (2) bacteria dislodged from the sediment and immediately plated, and (3) bacteria dislodged from the sediment, encapsulated in agarose, and then immediately plated.

## Materials and methods

### Collection and preparation of environmental bacteria

Intertidal sediment and near-shore seawater were collected from Charlottetown Harbour, PE (46.238 °N, 63.151 °W) in July (water temperature: 17.8°C; 24.4 ppt salinity; pH 8.11) using sterile Whirl-Pak.^®^ Sediment was separated into 10 ml aliquots and centrifuged at 4,500 *g* for 5 min and the excess seawater was decanted. Ten milliliters of diluted (1:10) Marine Broth; (dMB; BD Difco™, Fisher Scientific, United States) was added to each sediment sample, shaken by hand vigorously for 60 s then centrifuged at 4,500*g* for 5 min. The supernatant was decanted, and this process was repeated twice to remove excess salt water. To dislodge sediment-associated bacteria from sediment particles, 10 ml of dMB was added to the washed sediment sample and vortexed for 60 s. The tube was then shaken horizontally on a shaking platform at 400 rpm for 2 h and then vortexed for an additional 30 s. Large sediment particles were allowed to settle for 60 min at room temperature (RT) and the supernatant was transferred to a new tube. The concentration of bacteria in each sample was determined *via* fluorescent microscopy. Cells were stained using a live/dead bacterial viability kit (PromoCell, Germany) according to the manufacturer’s recommendations. Cells were counted using INCYTO C-Chip disposable hemocytometers (VWR, Radnor, PA) observed under a REVOLVE 4 upright inverted brightfield fluorescent microscope (Echo Laboratories, San Diego, CA) according to the manufacturer’s recommendations.

The volume necessary to attain ~8.35 × 10^6^ cells/ml was centrifuged at 4,500*g* for 10 min to concentrate the cells. Cells obtained from the sediment samples were processed in two ways. Cells were either resuspended in RT dMB by gentle aspiration or gently resuspended in 1% (w/v) low gelling temperature (LGT) agarose (gel strength >200 g/cm^2^_,_ melting temperature 26°C–30°C, RNase and DNase free; Sigma Canada; product. no. A9414) prepared in 10% dMB at 35°C and then encapsulated into 80 ± 20 μm diameter microbeads using a microfluidic chip as described previously (Alkayyali et al., 2021). Our previous research using model marine bacteria indicated that encapsulation using LGT agarose prepared in dMB at 35°C minimized the impact of the encapsulation process on viability (Pope et al., 2022).

### *In situ* incubation

To incubate processed samples in the environment from which the sediment was collected (*in situ* incubation) we utilized Slide-A-Lyzer™ gamma irradiated dialysis cassettes (Thermo Fisher, Canada). A membrane with a 10 kDA cut-off was chosen to retain bacteria but allow nutrients and small molecules to diffuse into the cassette. Prior to using the cassettes, we tested several strategies to aseptically load and unload the cassettes. Cassettes were loaded and unloaded using sterile 1 ml Luer-Lok™ syringes fitted with a sterile 21-gauge hypodermic needle (BD Biosciences, Canada). As the cassettes are sterilized by gamma-irradiation aseptic loading was readily achieved using the standard aseptic technique. After *in situ* incubation, we anticipated that the loading port would be colonized by marine bacteria and that the port would need to be decontaminated to prevent the introduction of external microorganisms into the contents of the cassette during unloading. Two disinfectants were tested for this purpose alone and in combination: 0.5% accelerated hydrogen peroxide (PREempt RTU™, Contec Inc., Canada) and 70% (v/v) isopropanol (Millipore Sigma, Canada). To test decontamination strategies, dialysis cassettes were loaded with sterile seawater and then placed in a 15-gallon aquarium containing a 5 cm bed of sediment and ~60 L of natural seawater and maintained at 22°C with aeration for 24 h. After incubation, the cassettes were unloaded after decontaminating the port and the contents were plated on Marine Agar (MA; BD Difco™, Fisher Scientific, United States). Contamination was assessed after incubation for 7 days at RT (22°C–25°C). Decontamination of the loading port using a sequential three-part process was determined to be the most reliable method and consisted of the following steps: (1) flushed the port with 0.5% (v/v) accelerated hydrogen peroxide followed by a 2 min contact time, (2) flushed the port with 70% (v/v) isopropanol followed by a 10 min contact time (3) flushed the port with sterile water to remove traces of isopropanol before inserting the needle.

To test the integrity of the dialysis cassette during extended incubation in a marine environment, cassettes were loaded with 0.5 ml of sterile water and then placed in the aquarium for 7 days after which the contents were aseptically removed and 100 μl aliquots plated on triplicate MA plates and observed for growth after 7 days at RT. The seal of unmodified dialysis cassettes routinely failed (i.e., microbial growth was observed on MA). Several clamping methods to reinforce the seal of the cassettes were evaluated using the above methodology (6 replicates/trial). The most effective clamping method was to use two pairs of galvanized mending plates (5.00 cm × 1.27 cm; Hillman, Canada) and stainless-steel nuts and bolts and to evenly apply pressure across the surface of the dialysis cassette.

For *in situ* incubation of the resuspended and encapsulated samples, dialysis cassettes were aseptically unpackaged in a biosafety cabinet and soaked in sterile dMB to hydrate the membranes. The cassettes were then loaded with 0.5 ml of either resuspended or encapsulated sediment bacteria using a sterile 1 ml syringe with a 21-gauge needle through one of the four loading ports according to the manufacturer’s recommendations. Six dialysis cassettes were loaded for each sediment treatment (i.e., resuspended or encapsulated). It should be noted that the inner diameter of the 21-gauge needle tip was 0.51 mm which was large enough to allow microbead injection without damaging the beads. Each dialysis cassette was then clamped with two pairs of galvanized mending plates and transported back to the collection site (~12 h after initial collection) at RT and gently buried beneath the sediment (~5 cm) in a nylon mesh bag to protect cassettes from damage from marine life. After 7 days the cassettes were gently removed from the sediment and transported back to the laboratory dry and at RT. Samples were processed immediately after collection from the environment. The exterior surface of each dialysis cassette was first washed with sterile dMB to remove excess debris. The removal port was decontaminated as previously described and a sterile syringe with a 21-gauge needle tip was then used to remove the contents of each dialysis cassette by inserting the needle tip through a port into the center of the dialysis cassette. The contents of each dialysis cassette were placed in a sterile tube for further analysis.

### Cell abundance, viability, and diversity assessment

To determine the effect of *in situ* incubation on both encapsulated and resuspended bacterial samples we assessed bacterial abundance, viability, and diversity before and after *in situ* incubation. To assess abundance, aliquots from encapsulated and resuspended samples, before and after incubation, were serially log-diluted and 100 μl aliquots of each dilution were spread on triplicate MA plates. The plates were incubated at RT for 5 days and the number of colonies observed on each plate was counted to determine colony forming units per mL (CFU/ml) for each sample.

Cell viability was assessed before and after incubation for both encapsulated and resuspended samples *via* a fluorescent Live/Dead bacterial staining kit as described previously (Alkayyali et al., 2021) with the modification that the resuspension buffer was changed from filter-sterilized seawater (FSSW) to dMB. This modification was made to reduce the fluorescent background observed in FSSW caused by marine ultramicrobacteria that are not removed by 0.22 μm filtration (Schut et al., 1993). To assess the effect of *in situ* incubation on the taxonomic composition in the resuspended and encapsulated samples. Assessment of the diversity within each sample was performed *via* random selection of colonies from triplicate plates for each set (*n* = 3). A minimum of 20 colonies were isolated from each sample by serial subculturing and purified isolates were identified by partial 16S rRNA gene sequencing. Template DNA for PCR was prepared by dispersing an isolated colony in 50 μl of dimethyl sulfoxide (DMSO; Millipore Sigma, Canada). The 16S rRNA gene was amplified using the pA (5′-AGAGTTTGATCCTGGCTCAG) and pH (5′-AAGGAGGTGATC CAGCC) primers as described previously (Duncan et al., 2015). Amplicons were directly sequenced at McGill University and Genome Quebec Innovation Centre using the 936R primer (5′-GTGCGGGCCCCCGTCAATT) using an ABI 3730xl DNA Analyzer. Sequences were analyzed in Geneious (Biomatters Inc., v 7.1) and clustered into operational taxonomic units (OTUs) using a 99% sequence identity cutoff value and a representative sequence was used for further analysis (Nichols et al., 2010). The taxonomic affiliation of the OTUs was inferred from a nucleotide search of the 16S rRNA database of GenBank using the Basic Local Alignment Search Tool (BLAST; Zhang et al., 2000). Partial 16S rRNA gene sequences representing OTUs were deposited in GenBank under accession numbers ON385874 to ON385929.

The diversity of each sample was assessed using the Species-richness and Diversity Estimation in R program (SpadeR; Chao and Chiu, 2016). Species richness and diversity were calculated for each sediment treatment. Estimated richness was calculated using the Choa1 estimator (Chao and Chiu, 2016). The Shannon and Simpson diversity indices were calculated within SpadeR and the indices calculated using the maximum likelihood method are reported. The 95% confidence intervals calculated by SpadeR are provided for the Chao1 estimator and diversity indices (Chao, 1984).

### Statistics

All statistical analyses were performed using GraphPad Prism 8 (La Jolla, California, United States). Statistical significance was determined using unpaired, two-tailed *t*-tests and the Holm-Sidak method of correction for *p*-values, with an alpha of 0.05. Each data set was analyzed individually without assuming a consistent standard deviation.

## Results and discussion

### Dialysis cassette modifications

Commercially available Slide-A-Lyzer dialysis cassettes were chosen as vessels for *in situ* incubation experiments conducted in this study because they presented a readymade diffusion chamber that would retain bacteria encapsulated in microbeads while the dialysis membrane would prevent infiltration of external microorganisms into the chamber and allow nutrients to diffuse across the membrane. The 10 kDa dialysis cassettes could be purchased gamma-irradiated providing the sterility required for microbiology studies. Initial testing of the cassettes in a saltwater aquarium over 7 days determined that the seal failed after 2 days, allowing external bacteria into the chamber. This was undesirable as infiltration of external bacteria into the chamber would overwhelm the growth of encapsulated bacteria and confound the study results. Rather than applying adhesives to the cassette, which might present biocompatibility issues, we tested a variety of physical clamping mechanisms to reinforce the cassette seal. The most effective method was the use of small mending plates that were placed across the cassette and held in place with stainless steel bolts and nuts. This method proved to be an effective, simple, and reproducible method of guaranteeing the integrity of the cassette seal during incubation periods of up to 7 days. Another issue encountered when using the dialysis cassettes was microbial fouling of the injection ports during incubation. We tested disinfectants and found that both accelerated hydrogen peroxide (PREempt RTU; 0.5% H_2_O_2_) and 70% IPA were not effective individually, however when we combined the two disinfectants in a sequential process we achieve reproducible sanitization of the injection port, allowing for the aseptic removal of the contents of the dialysis chamber.

### Cell abundance and viability assessment

**Figure 1:**
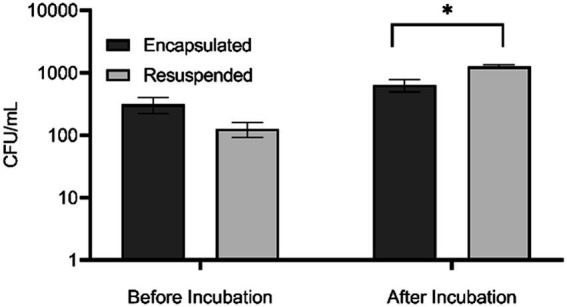
Abundance of bacteria in encapsulated and resuspended marine sediment bacteria samples before and after *in situ* incubation based upon colony counts performed in triplicate. Error bars indicate standard error, ^*^ denotes significance of *p* < 0.01. Is this correct?

To evaluate the effect of *in situ* incubation on recoverable bacterial diversity we compared four treatments. First, we prepared microorganisms in two ways. Bacteria dislodged from a marine sediment sample were concentrated by centrifugation and either encapsulated in agarose microbeads or resuspended in dMB. The “encapsulated” and “resuspended” samples were then split, and one portion was plated immediately on agar plates while the other portions were injected into dialysis cassettes and then incubated *in situ* in the environment for 7 days. To assess the effect of these different treatments on bacterial abundance, colony counts were performed. The abundance of culturable bacteria in resuspended and encapsulated bacteria prior to *in situ* incubation was not significantly different (*t*(16) = 1.93, *p* = 0.0728). This result indicated that the encapsulation process did not adversely affect the viability of cells, confirming our previous results using model marine bacteria (Alkayyali et al., 2021). After *in situ* incubation, the abundance of bacteria obtained from the resuspended samples was significantly greater than that of the encapsulated samples (*t*(7) = 2.92, *p* = 0.0452; Figure 1). The lower bacterial abundance in the encapsulated sample may be due to a slower growth rate of encapsulated bacteria compared to non-encapsulated bacteria due to the requirement of oxygen and nutrients to diffuse into the agarose bead to be utilized by the bacteria. It may also be because the encapsulated bacteria formed aggregates in the agarose matrix that affected dispersion during plating, resulting in a lower apparent abundance. Future experiments could assess the growth rates of encapsulated bacteria under varying nutrient and oxygen concentrations to determine if diffusion rates limit growth. In addition, hypoxia sensing molecular probes (e.g., Image-iT™ Green Hypoxia Reagent, Fisher Scientific) could be used to assess oxygen limitations in beads. Thorough homogenization of beads prior to plating could also provide a more accurate assessment of abundance of colonies that develop in agar microbeads.

**Figure 2:**
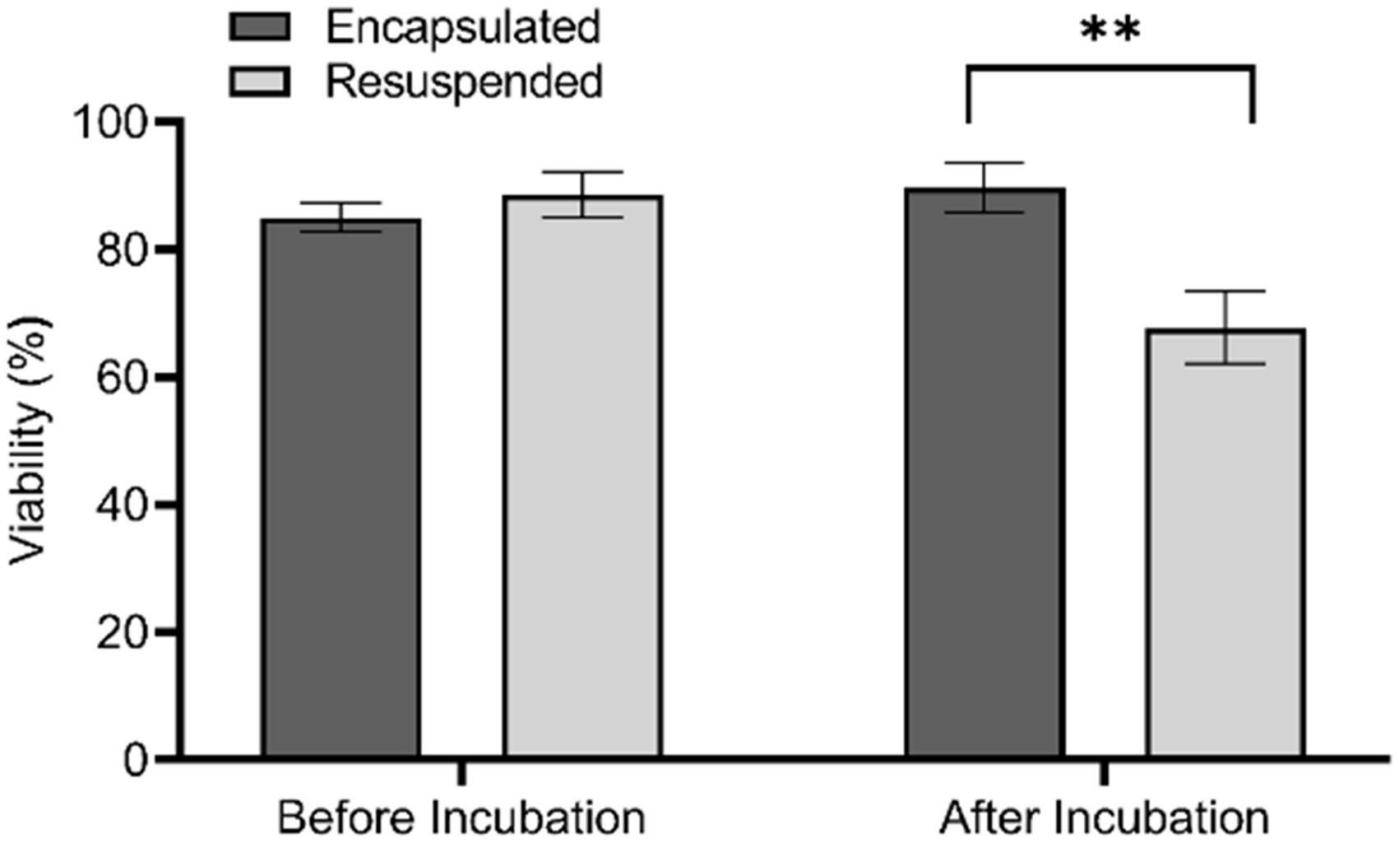
Cell viability for encapsulated and resuspended marine sediment bacteria samples before and after *in situ* incubation based upon a Live/Dead assay. Error bars indicate standard error, ^**^ denotes significance of *p* ≤ 0.01.

To assess the effect of the different treatment methods on bacterial viability we used a fluorescent Live/Dead assay to microscopically determine the percentage of viable cells present in each sample. This assay stains viable cells green while dead cells appear red. Prior to *in situ* incubation, the proportion of viable cells present in encapsulated and resuspended samples was not significantly different (*t*(24) = 0.87, *p* = 0.399). Conversely, there was a significantly greater proportion of viable of cells within encapsulated samples compared to resuspended samples after *in situ* incubation (*t*(25) = 3.23, *p* = 0.0035; Figure 2). The increased viability of encapsulated samples following incubation indicates that although the abundance determined by colony counts on agar plates demonstrates a lower abundance compared to non-encapsulated samples, there are in fact a greater proportion of viable cells within these samples.

**Figure 3:**
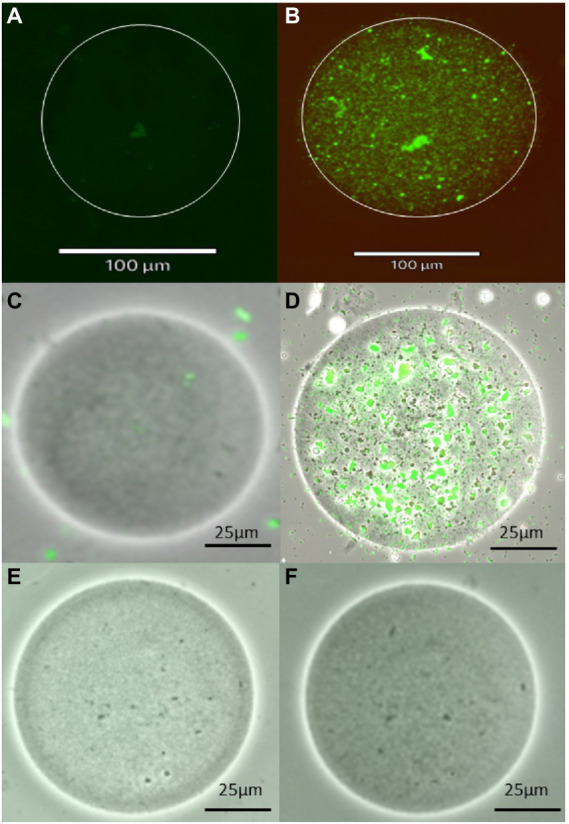
Images of agarose microbeads, including a microbead containing a single marine sediment bacterial cell before incubation **(A,C)** and the growth of numerous cells within the agarose microbead following incubation **(B,D)** using fluorescent and brightfield imaging, respectively as well as images of blank beads without any bacteria **(E,F)**. Images were obtained using Live/Dead bacterial stain and Revolve4 microscope with a 20× objective lens.

The images of encapsulated bacteria before and after incubation provide evidence of the growth of microencapsulated bacteria (Figure 3). Prior to incubation, single colonies or a few individual colonies were observed within the microbeads while after incubation, microbeads contained increased growth of colonies. The bacteria appeared to form microcolonies within the agar beads, which might explain why the bacterial abundance in plate counts of *in situ* incubated encapsulated samples appeared to be lower. Interestingly, the percent viability of encapsulated cells before and after incubation was not significantly different, while the percent viability of resuspended samples was significantly lower after incubation. The reason for this is unknown, but it is possible that the non-encapsulated samples were able to proliferate more quickly than encapsulated bacteria, resulting in a greater number of cells that had entered the death phase. This hypothesis is supported by the data in Figure 1, which shows that the total number of cultivable cells in the resuspended sample following *in situ* incubation was greater than that of the encapsulated sample. Alternatively, it might suggest that agarose encapsulation may protect encapsulated cells from toxic chemicals present in the environment, such as tannins derived from organic matter present in the sediment (Kaczmarek, 2020), or from antimicrobial metabolites produced by microorganisms in the sediment or even within the diffusion chamber (Thomashow et al., 1990).

### Diversity assessment

A primary motivation for this study was to develop a new cultivation method to enable the cultivation of a greater biodiversity of the microbiome present in environmental samples such as marine sediments and soils, as unique bacteria from these habitats have previously been shown to be an excellent source of metabolites with biomedical potential (Gulder and Moore, 2010; Ling et al., 2015). To assess the diversity of bacteria cultured from each of the four treatments, randomly selected colonies from each treatment were purified and 148 isolates (32–38 isolates from each treatment; Supplementary Table 1) were identified. A total of 58 OTUs were obtained using a 99% sequence identity threshold and 8–27 OTUs were obtained from each treatment (Table 1). The number of OTUs was similar between the encapsulated and resuspended samples both before and after incubation, although in both cases fewer OTUs were detected in the resuspended samples (Table 1). The observed richness also decreased following *in situ* incubation in both the encapsulated and resuspended treatments. Similar trends were observed in the Choa1 richness estimator and Shannon and Simpson diversity indices (Table 1), indicating that both species richness and diversity decreased after *in situ* incubation.

**Table 1:** Summary of the diversity obtained from two sediment treatments before and after *in-situ* incubation.

	Before incubation		After incubation	
	Encapsulated	Resuspended	Encapsulated	Resuspended
Sample size (*N*)	38	32	38	38
Richness (OTUs)	27	23	14	8
Est.Richness (Choa1) [95% CI]	98.6 [46.8–285.9]	75.3 [37.0–219.0]	29.6 [16.9–96.6]	12.4 [8.5–47.9]
Shannon Diversity [95% CI]	23.3 [16.4–30.3]	19.7 [13.6–25.8]	8.7 [5.2–12.3]	5.3 [3.7–6.9]
Simpson Diversity [95% CI]	19.5 [11.9–27.2]	16.0 [9.3–22.7]	5.8 [3.4–8.3]	4.3 [3.0–5.6]
Identity >98.7% (# isolates)	23	18	30	37
Identity >97–98.7 (# isolates)	8	13	4	0
Identity >95–<97% (# isolates)	3	1	4	1
Identity 93–<95% (# isolates)	4	0	0	0

OTUs were defined as sharing >99% sequence identity. Estimated richness (Choa1 index)(38) and Shannon and Simpson diversity indices were calculated using SpadeR.(37) The number of OTUs from each sample exhibiting a range of sequence identities to 16S rRNA gene sequences of type strains contained in the GenBank 16S rRNA database is provided in the bottom four rows.

**Figure 4:**
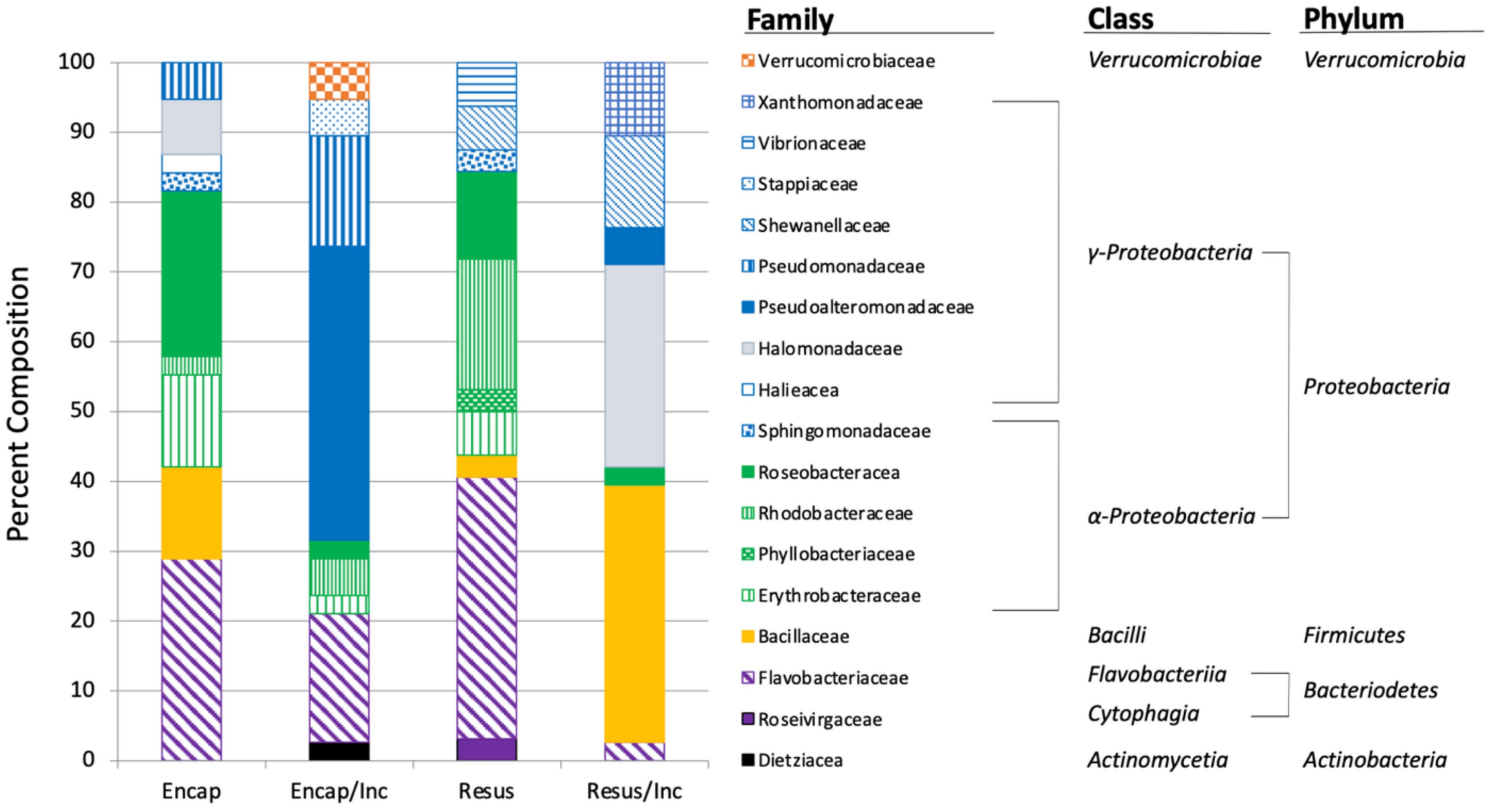
Taxonomic composition of bacteria isolated from marine sediment using four treatments: Encap: encapsulated, Encap/Inc. – encapsulated and incubated *in situ*, Resus – resuspended, Resus/Inc. – resuspended and incubated *in situ*. The bar graph shows family-level taxonomic classification.

In light of this result, we were interested to assess the distribution of OTUs obtained from the four treatments. Pairwise comparisons of OTUs shared between the four samples revealed that there was a low degree of overlap (Figure 4). No OTU was shared between more than two treatments and, as might be expected, the greatest overlap was between the encapsulated and resuspended samples prior to *in situ* incubation (6 OTUs). Only three OTUs were in common between the two encapsulated samples and no OTUs were found in common between the two resuspended samples. This low degree of shared OTUs between samples both before and after *in situ* incubation is somewhat surprising and may be partially due to the limited number of isolates sampled from each treatment. This result also suggests that the different treatments should be selected for the growth of a different subset of the microbial community.

**Figure 5:**
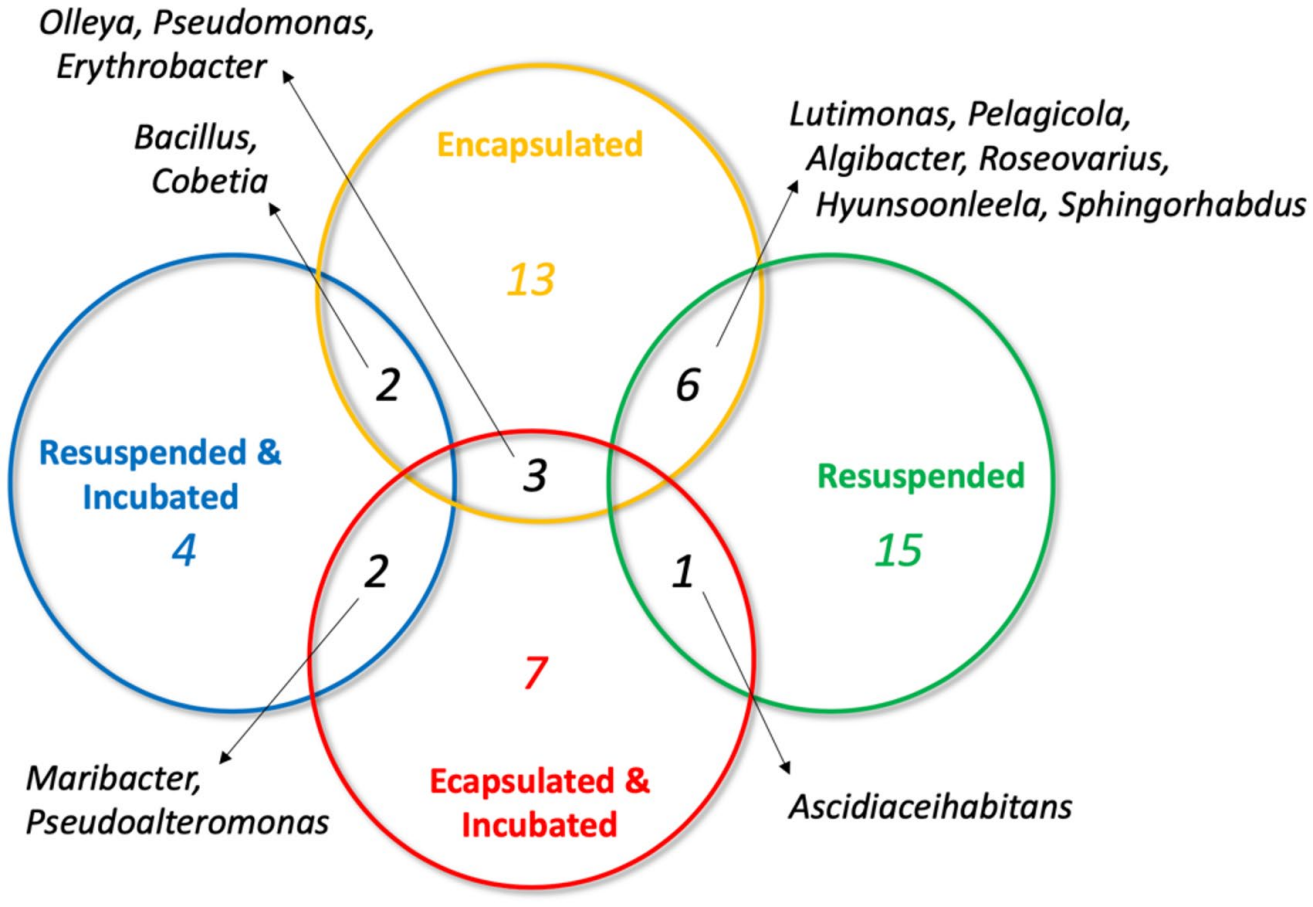
Venn diagram illustrating the distribution of shared OTUs between the four sediment treatments. Genera represented by the OTUs are indicated.

Taxonomic classification of the isolates revealed that *Proteobacteria*, *Bacteroidetes,* and *Firmicutes* were identified in all treatments with the majority of isolates (56.3%–75.7%) recovered from each treatment belonging to the *Proteobacteria* (Figure 5). The dominance of *Proteobacteria* is consistent with previous studies examining microbial diversity of marine sediments and seawater (Hoshino et al., 2020). *Actinobacteria* and *Verrucomicrobia* were only obtained from samples that had been encapsulated and incubated *in situ* (Figure 5). The inability to culture these taxa from samples that were plated immediately may have been because they were so scarce in the environmental samples that they were not represented in the sub-samples that were plated, or because they were incapable of growing on agar media. The process of encapsulation and *in situ* incubation may have enabled the recovery of these taxa by fostering their growth to levels that were detectable when plated after *in situ* incubation. The process may also have acclimatized the bacteria to growing on an agar matrix, allowing them to form colonies when they were plated after *in situ* incubation. In other words, the bacteria were effectively domesticated for cultivation in the laboratory. Interestingly, Nichols and colleagues (Nichols et al., 2010) also only detected *Verrucomicrobia* from in samples that had been embedded in agar and incubated *in situ* in their description of the Ichip. The OTU (OTU24, Supplementary Table 1) belonging to the *Verrucomicrobia* was comprised of two isolates whose 16S rRNA gene sequence exhibited only 95.85% identity to *Luteolibacter algae* (NR_041624), suggesting this OTU represents a novel species of *Luteolibacter. Luteolibacter* has been isolated from a variety of habitats including marine habitats (Zhang and Demain, 2005; Yoon et al., 2008; Dahal et al., 2021), and recently, *Luteolibacter gellanilyticus* was formally described after being isolated from forest soil using an Ichip diffusion chamber (Pascual et al., 2017). These results indicate that our methodology provides similar access to difficult-to-culture microbiota that has previously been accessed using the Ichip diffusion chamber design.

At the family level of taxonomic resolution, the differences in microbiota recovered from the four treatments become more apparent (Figure 5). For example, *Flavobacteriaceae* represented 18.5%–37.5% of the communities of three treatments, but only 2.7% of the community recovered from the resuspended/incubated treatment. Conversely, *Bacillaceae* were a major component of the resuspended/incubated treatment (36.7%) but accounted for a much smaller proportion (0%–13.2%) in the other treatments. One of the most striking differences was the large percentage of isolates belonging to the *Pseudoalteromonadaceae* obtained from the encapsulated/incubated (42.1%) and resuspended/incubated (5.3%) treatments, but completely absent in the other treatments. The *Pseudoalteromonadaceae* component of this community was composed of two OTUs (OTU2 and OTU13) that exhibited close 16S rRNA gene sequence similarity to *Pseudoalteromonas tetraodonis* (NR_114187, 100% identity) and *Pseudoalteromonas shioyasakiensis* (NR_125458, 98.8% identity). Both strains are marine bacteria that require salt for growth (Ivanova et al., 2001; Matsuyama et al., 2014). Although the type strains of these species were not isolated using diffusion chambers, it is tempting to hypothesize that their recovery in the *in situ* incubated treatments was facilitated by the combination of encapsulation and *in situ* incubation in a marine environment. Comparing samples before and after *in situ* incubation, there were also several instances where taxa present prior to incubation were not recovered after incubation (e.g., *Bacillaceae* in the encapsulated samples and *Roseivirgaceae* in the resuspended samples). This may have been due to members of these taxa being reduced in relative abundance due to the more rapid replication of other taxa whose growth was favored by *in situ* incubation. Examining a larger sample size from each treatment may have reduced the incidence of this occurrence by increasing the probably of recovering taxa whose relative abundance decreased during *in situ* incubation.

To assess the ability of the four treatments to recover novel taxa from marine sediment, we compared the number of isolates recovered from each treatment that exhibited different levels of 16S rRNA gene sequence identity to sequences of type strains in GeneBank. Thresholds of 98.7 and 95% identity were used as species and genus-level cut-offs, respectively (Stackebrandt, 2006); however, it should be noted that these thresholds are generalized cutoffs and do not apply to all bacterial taxa (Rossi-Tamisier et al., 2015). To provide further discrimination between the taxonomic novelty of strains obtained from the different treatments we also used two arbitrary sequence identity categories (>97%–98.65%, and >95%–<97%; Table 1). The recovered isolates from the resuspended/incubated sample presented the least taxonomic novelty as 97% of isolates exhibited >98.7% sequence identity with previously described bacterial species. A larger number of taxonomically unique isolates was obtained from the resuspended sample that was directly plated, with 56% of the isolates showing >98.7% identity, 41% showing 97%–98.7% identity, and 3% showing 95%–97% identity to previously described bacteria. In contrast, a much larger proportion of strains isolated from the encapsulated samples exhibited sequence identities <97% to described bacteria (encapsulated – 18%, encapsulated/incubated 11%). Interestingly, fewer potentially novel species were recovered from the encapsulated samples after *in situ* incubation as only 22% of isolates exhibited <98.65% identity to described species compared to 49% from the encapsulated and plated sample. The encapsulated sample prior to *in situ* incubation was also the only treatment to recover novel bacterial genera exhibiting <95% sequence identity to described bacteria.

Overall, the results of this study revealed trends where encapsulated samples exhibited greater richness, diversity, and taxonomic novelty than resuspended samples, while *in situ* incubation reduced the richness, diversity, and taxonomic novelty. Microencapsulation alone may increase the microbial diversity that can be recovered from marine sediments by providing nutrients, a scaffold for growth, and protection from external hazards. Agarose utilization is broadly distributed in marine bacteria (Hehemann et al., 2014; Lombard et al., 2014) and the agarose was supplemented with dMB, thus it is likely that the provision of nutrients contributed to the improved recovery observed from the encapsulated treatments. A similar observation was made by Kaeberlein and colleagues in their seminal report on the use of diffusion chambers (Kaeberlein et al., 2002). Agarose encapsulation has also been used to maintain the viability of bacteria exposed to harsh environmental conditions (Alehosseini et al., 2019), thus the agarose may have protected the viability of sensitive encapsulated bacteria until they reached a cell density where colonies could be established on agar plates. Another explanation for positive effect of encapsulation on recoverable microbial diversity could be due to the exposure of cells to a mild heat shock during the encapsulation process, which could resuscitate viable but non-culturable cells (Li et al., 2014).

Unexpectedly, *in situ* incubation had a negative effect on the diversity and taxonomic novelty of microbes recovered from the sediment sample. This observation is contrary to results obtained in previous studies utilizing combinations of agarose encapsulation and a variety of enrichment or *in situ* incubation techniques (Ji et al., 2012; Ma et al., 2014). Explanations for this difference may be the short duration (7 days) of *in situ* incubation and the use only a single round of *in situ* incubation. *In situ* incubation periods for diffusion-chamber-based studies have ranged from 2 to 4 weeks and the contents of the chambers have been subjected to multiple iterations of *in situ* incubation (Bollmann et al., 2007; Nichols et al., 2010; Steinert et al., 2014). The convenient format of the dialysis cassettes makes aseptic sample recovery very straightforward, thus future studies could readily assess the effect of multiple *in situ* incubation periods. Our study also utilized a different membrane pore size than previous studies, which typically used membranes with 30–200 nm pores (Bollmann et al., 2007; Nichols et al., 2010; Steinert et al., 2014). The 10 kDa membranes used in this study correspond to a pore size of 3 nm, which is an order of magnitude smaller than the membranes used previously. It is possible that this small pore size restricted the diffusion of macromolecular nutrients (e.g., proteins and polysaccharides) into the diffusion chamber and limited the growth of the bacteria in the chambers.

The objective of many biotechnology-focused bioprospecting efforts is to culture as broad a selection of bacterial taxa as possible so that they can be interrogated for a variety of traits of biotechnological interest. These efforts are understandably focused on culturing novel taxa, which are more likely to possess novel traits than commonly cultured bacteria. Diffusion-chamber-based approaches have already delivered these novel discoveries (Ling et al., 2015; MacIntyre et al., 2019) and will continue to do so into the future as their use becomes more common. In this study, the taxonomic diversity of bacteria recovered from a marine sediment sample was highly dependent on the treatment that the sediment was subjected to. While the small sample size of the study limits the conclusions that can be drawn, the results showed that minimal taxonomic overlap was observed in the bacteria recovered in a random selection from the different treatments (Figure 4). This trend suggests that the use of multiple isolation strategies is key to maximizing the recovery of the viable but uncultured majority of environmental bacteria. The methodology we report here provides an easily implementable approach to improve the diversity of bacteria recovered from environmental samples using microencapsulation and *in situ* incubation of dialysis cassette diffusion chambers.

## Conclusion

Based upon assessments of the abundance, viability, and diversity, microencapsulation of marine sediment bacteria led to the recovery of greater taxonomic diversity of bacteria than the traditional method of simply plating bacteria directly from the environmental sample. As a large proportion of environmental bacteria live attached to surfaces in their natural environment, the agar microbeads likely benefit the growth of difficult-to-culture bacteria providing a surface for growth, nutrients, and protection from stressors (Alain and Querellou, 2009; MacIntyre et al., 2019). Combining microencapsulation with *in situ* incubation further enhanced the recoverable microbial diversity by exposing the bacteria to natural environmental conditions including chemical signaling that is not well understood. The *in situ* method described here employs commercially available dialysis cassettes that can be easily obtained from a myriad of scientific suppliers, making the method readily available to researchers. Further improvements in the *in situ* incubation process can be realized by exploring different membrane pore sizes. The use of microencapsulation technology also makes high throughput downstream processing techniques such as flow cytometry or fluorescent activated cell sorting (FACS) to facilitate the rapid isolation of pure strains after *in situ* incubation possible. Importantly, the use of the new bacterial cultivation method described here has resulted in the isolation of several putatively new species of bacteria and the further use and improvement of this method will provide access to many novel bacteria that can be explored for their biotechnological potential.

## Data availability statement

The datasets presented in this study can be found in online repositories. The names of the repository/repositories and accession number(s) can be found in the article/Supplementary material.

## Author contributions

EP, CC, BH, RK, and AA: conceptualization and writing– review and editing. EP, CC, and BH: data curation. EP and BH: formal analysis. RK and AA: funding acquisition, project administration, and resources. EP: investigation, validation, visualization, and writing–original draft. EP and CC: methodology. BH, RK, and AA: supervision. All authors contributed to the article and approved the submitted version.

## Funding

This work was supported by the NSERC Discovery Grant (RGPIN-2017-05272), Canada Research Chair program, New Frontiers in Research Fund (NFRFE-2018-01410), and Canada Foundation for Innovation Grant (Project #37696).

## Conflict of interest

The authors declare that the research was conducted in the absence of any commercial or financial relationships that could be construed as a potential conflict of interest.

## Publisher’s note

All claims expressed in this article are solely those of the authors and do not necessarily represent those of their affiliated organizations, or those of the publisher, the editors and the reviewers. Any product that may be evaluated in this article, or claim that may be made by its manufacturer, is not guaranteed or endorsed by the publisher.
