# Supplementary material for: Microencapsulation and in situ incubation methodology for the cultivation of marine bacteria
Source: Front Microbiol. 2022 Aug 22;13:958660. doi: 10.3389/fmicb.2022.958660 (PMC9441948; doi:10.3389/fmicb.2022.958660)
Supplement: Supplementary file 1 [file Table_1.DOCX]

Supplementary Material

**Microencapsulation and *In-Situ* Incubation Methodology for the Cultivation of Marine Bacteria**

Emily Pope^1^, Christopher Cartmell^2^, Bradley Haltli^1,3^, Ali Ahmadi^1,4,5^, and Russell G. Kerr^1,2,3*^

^1^Department of Biomedical Science, University of Prince Edward Island, Charlottetown, PE, Canada

^2^Department of Chemistry, University of Prince Edward Island, Charlottetown, PE, Canada

^3^Nautilus Biosciences Croda, Charlottetown, PE, Canada

^4^Faculty of Sustainable Design Engineering, University of Prince Edward Island, Charlottetown, PE, Canada

^5^Department of Mechanical Engineering, École de technologie supérieure (ÉTS), Montreal, QC, Canada

*** Correspondence:**Russell G. Kerr
rkerr@upei.ca

# Supplementary Data

Supplementary Table 1: Summary of OTU distribution from two sediment treatments (encapsulated – “Encap” and resuspended – “Resus”) before and after *in-situ* incubation (“Inc”). A representative sequence for each OTU is provided as well as the GenBank accession number of the deposited sequence. The closest match from a query of the GenBank 16S rRNA database is indicated as well the percent identity of the sequence match.

|  |  |  | **OTU Abundance** | | | |  |  |
| --- | --- | --- | --- | --- | --- | --- | --- | --- |
| **OTU** | **Colony code** | **GeneBank Accession Number** | **Encap** | **Encap/Inc** | **Resus** | **Resus/Inc** | **Closest BlastN Match (GenBank Accession #)** | **Identity (%)** |
| 1 | RKEP0137 | ON385874 | 4 | 0 | 0 | 13 | *Bacillus mycoides* (NR_024697.1) | 99.89 |
| 2 | RKEP0115 | ON385875 | 0 | 13 | 0 | 0 | *Pseudoalteromonas tetraodonis* (NR_114187.1) | 100.00 |
| 3 | RKEP0051 | ON385876 | 1 | 0 | 0 | 11 | *Cobetia pacifica* (NR_113402.1) | 99.88 |
| 4 | RKEP0016 | ON385877 | 0 | 2 | 0 | 0 | *Roseibium marinum* (NR_043040.1) | 98.05 |
| 5 | RKEP0024 | ON385878 | 2 | 6 | 0 | 0 | *Pseudomonas oceani* (NR_152090.1) | 99.65 |
| 6 | RKEP0011 | ON385879 | 4 | 1 | 0 | 0 | *Erythrobacter aquimaris* (NR_025789.1) | 98.38 |
| 7 | RKEP0067 | ON385880 | 0 | 0 | 1 | 0 | *Erythrobacter aquimaris* (NR_025789.1) | 98.46 |
| 8 | RKEP0059 | ON385881 | 1 | 0 | 5 | 0 | *Lutimonas saemankumensis* (NR_044441.1) | 99.65 |
| 9 | RKEP0028 | ON385882 | 1 | 4 | 0 | 0 | *Olleya marilimosa* (NR_116100.1) | 99.88 |
| 10 | RKEP0175 | ON385883 | 3 | 0 | 2 | 0 | *Pelagicola litorisediminis* (NR_118566.1) | 99.87 |
| 11 | RKEP0078 | ON385884 | 0 | 0 | 0 | 5 | *Shewanella baltica* (NR_025267.1) | 98.87 |
| 12 | RKEP0142 | ON385885 | 0 | 0 | 0 | 4 | *Stenotrophomonas rhizophila* (NR_121739.1) | 100.00 |
| 13 | RKEP0116 | ON385886 | 0 | 3 | 0 | 2 | *Pseudoalteromonas shioyasakiensis* (NR_125458.1) | 98.82 |
| 14 | RKEP0173 | ON385887 | 0 | 1 | 3 | 0 | *Ascidiaceihabitans donghaensis* (NR_133970.1) | 97.97 |
| 15 | RKEP0190 | ON385888 | 1 | 0 | 1 | 0 | *Sphingorhabdus flavimaris* ( NR_025814.1) | 99.63 |
| 16 | RKEP0186 | ON385889 | 0 | 0 | 1 | 0 | *Lysinibacillus massiliensis* (NR_043092.1) | 100.00 |
| 17 | RKEP0060 | ON385890 | 0 | 0 | 2 | 0 | *Shewanella donghaensis* (NR_042846.1) | 98.14 |
| 18 | RKEP0053 | ON385891 | 1 | 0 | 1 | 0 | *Algibacter agarilyticus* (NR_118327.1) | 96.58 |
| 19 | RKEP0113 | ON385892 | 0 | 1 | 0 | 1 | *Maribacter aestuarii* (NR_109501.1) | 96.43 |
| 20 | RKEP0174 | ON385893 | 1 | 0 | 1 | 0 | *Hyunsoonleella udonensis* (NR_145941.1) | 100.00 |
| 21 | RKEP0170 | ON385894 | 2 | 0 | 0 | 0 | *Pelagicola litorisediminis*(NR_118566.1) | 98.50 |
| 22 | RKEP0182 | ON385895 | 0 | 0 | 2 | 0 | *Yoonia rosea* (NR_043098.1) | 99.24 |
| 23 | RKEP0044 | ON385896 | 1 | 0 | 1 | 0 | *Roseovarius aestuarii* (NR_044424.1) | 99.75 |
| 24 | RKEP0029 | ON385897 | 0 | 2 | 0 | 0 | *Luteolibacter algae* (NR_041624.1) | 95.85 |
| 25 | RKEP0047 |  | 1 | 0 | 0 | 0 | *Bacillus pumilus* (NR_118381.1) | 99.64 |
| 26 | RKEP0158 | ON385898 | 2 | 0 | 0 | 0 | *Halomonas meridiana* (NR_042066.1) | 100.00 |
| 27 | RKEP0015 | ON385899 | 0 | 1 | 0 | 0 | *Dietzia cinnamea* (NR_042390.1) | 99.87 |
| 28 | RKEP0027 | ON385900 | 0 | 1 | 0 | 0 | *Actibacter sediminis* (NR_044349.1) | 98.80 |
| 29 | RKEP0033 |  | 0 | 1 | 0 | 0 | *Polaribacter porphyrae* (NR_114321.1) | 96.32 |
| 30 | RKEP0035 | ON385901 | 1 | 0 | 0 | 0 | *Muricauda aquimarina* (NR_042909.1) | 96.48 |
| 31 | RKEP0046 | ON385902 | 1 | 0 | 0 | 0 | *Zeaxanthinibacter enoshimensis* (NR_114017.1) | 100.00 |
| 32 | RKEP0052 | ON385903 | 1 | 0 | 0 | 0 | *Roseobacter ponti* (NR_157654.1) | 97.24 |
| 33 | RKEP0054 | ON385904 | 0 | 0 | 1 | 0 | *Tateyamaria armeniaca* (NR_169471.1) | 98.62 |
| 34 | RKEP0056 | ON385905 | 0 | 0 | 1 | 0 | *Eudoraea adriatica* (NR_042628.1) | 97.46 |
| 35 | RKEP0058 | ON385906 | 0 | 0 | 1 | 0 | *Fabibacter pacificus* (NR_109732.1) | 97.39 |
| 36 | RKEP0066 | ON385907 | 0 | 0 | 1 | 0 | *Vibrio alginolyticus* (NR_122059.1) | 99.75 |
| 37 | RKEP0189 | ON385908 | 0 | 0 | 1 | 0 | *Erythrobacter citreus* (NR_028741.1) | 97.88 |
| 38 | RKEP0070 | ON385909 | 0 | 0 | 0 | 1 | *Shimia sagamensis* (NR_137204.1) | 99.49 |
| 39 | RKEP0081 | ON385910 | 0 | 0 | 0 | 1 | *Planococcus maritimus* (NR_025247.1) | 99.31 |
| 40 | RKEP0109 | ON385911 | 0 | 0 | 1 | 0 | *Maribacter dokdonensis* (NR_043294.1) | 99.17 |
| 41 | RKEP0117 | ON385912 | 0 | 1 | 0 | 0 | *Tritonibacter mobilise* (NR_116522.1) | 99.87 |
| 42 | RKEP0159 | ON385913 | 1 | 0 | 0 | 0 | *Oceanicella actignis* (NR_118425.1) | 94.43 |
| 43 | RKEP0160 | ON385914 | 1 | 0 | 0 | 0 | *Muricauda ochracea* (NR_174330.1) | 94.15 |
| 44 | RKEP0167 | ON385915 | 1 | 0 | 0 | 0 | *Maribacter sedimenticola* (NR_025748.1) | 99.65 |
| 45 | RKEP0168 | ON385916 | 1 | 0 | 0 | 0 | *Tateyamaria pelophila* (NR_114961.1) | 98.93 |
| 46 | RKEP0171 | ON385917 | 1 | 0 | 0 | 0 | *Erythrobacter nanhaisediminis* (NR_116764.1) | 98.58 |
| 47 | RKEP0176 | ON385918 | 0 | 0 | 1 | 0 | *Maribacter antarcticus* (NR_044515.1) | 98.43 |
| 48 | RKEP0177 | ON385919 | 0 | 0 | 1 | 0 | *Winogradskyella pulchriflava* (NR_109526.1) | 99.43 |
| 49 | RKEP0178 | ON385920 | 0 | 0 | 1 | 0 | *Vibrio breoganii* (NR_044304.1) | 98.30 |
| 50 | RKEP0179 | ON385921 | 0 | 0 | 1 | 0 | *Loktanella acticola* (NR_158144.1) | 99.12 |
| 51 | RKEP0180 | ON385922 | 0 | 0 | 1 | 0 | *Aquimarina versatilis*(NR_156070.1) | 97.76 |
| 52 | RKEP0187 | ON385923 | 0 | 0 | 1 | 0 | *Hoeflea halophila* (NR_108835.1) | 99.74 |
| 53 | RKEP0012 | ON385924 | 0 | 1 | 0 | 0 | *Primorskyibacter* *sedentarius* (NR_113142.1) | 100.00 |
| 54 | RKEP0038 | ON385925 | 1 | 0 | 0 | 0 | *Shimia aestuarii* (NR_042903.2) | 99.37 |
| 55 | RKEP0039 | ON385926 | 1 | 0 | 0 | 0 | *Robiginitalea sediminis* (NR_164893.1) | 93.10 |
| 56 | RKEP0041 | ON385927 | 1 | 0 | 0 | 0 | *Winogradskyella echinorum* (NR_044564.1) | 94.82 |
| 57 | RKEP0042 | ON385928 | 1 | 0 | 0 | 0 | *Winogradskyella echinorum* (NR_044564.1) | 100.00 |
| 58 | RKEP0156 | ON385929 | 1 | 0 | 0 | 0 | *Marimicrobium arenosum* (NR_148595.1) | 95.20 |
